# Tick Bite Granuloma After Incomplete Removal of *Ixodes ricinus* Tick

**DOI:** 10.3390/insects16040389

**Published:** 2025-04-06

**Authors:** Katarzyna Bartosik, Agata Szczecina, Agnieszka Borzęcka-Sapko, Magdalena Raszewska-Famielec, Alicja Buczek

**Affiliations:** 1 Department of Biology and Parasitology, Chair of Pharmacology and Biology, Faculty of Health Sciences, Medical University of Lublin, Radziwiłłowska 11 St., 20-080 Lublin, Poland; alicja.buczek@umlub.pl; 2 Med-Laser Non-Public Health Care Centre, 20-406 Lublin, Poland; lek.agataszczecina@gmail.com (A.S.); agnieszka.borzecka@op.pl (A.B.-S.); 3 Faculty of Phisical Education and Health, University of Physical Education, 21-500 Biała Podlaska, Poland; raszewska.famielec@gmail.com

**Keywords:** *Ixodes ricinus*, tick bite granuloma, skin lesion, incomplete tick removal

## Abstract

*Ixodes ricinus* (Acari: Ixodidae) ticks infest humans in Europe most frequently. This report describes an interesting case of a persistent inflammatory skin reaction developed in a patient after the incomplete removal of an *I. ricinus* female tick. A 47-year-old female patient incompletely removed a female *I. ricinus* tick feeding in the lumbar region for approximately 12 h. After 9 weeks of persistent itching and burning, the skin from the tick feeding site was sampled for histopathological examination. The lesion exhibited the presence of abundant inflammatory infiltrates composed of lymphocytes, plasma cells, and eosinophils, as well as multinucleated macrophages and irregular fibrosis. The histopathological image revealed the presence of a tick bite granuloma. This study indicates that persistent symptoms of an inflammatory reaction may develop after incomplete tick removal, even after a short time of feeding.

## Simple Summary

Ticks are widespread hematophagous arthropods that feed on humans and animals. The species *Ixodes ricinus* is the most common tick that infests humans in Europe. A case of persistent inflammatory reaction that developed in a patient after the incomplete removal of an *I. ricinus* tick is described herein. A 47-year-old female partially removed a castor bean tick feeding on her back in the lumbar region. After nine weeks of constant itching and burning sensations, the patient had skin samples taken from the tick bite site for histopathological examination. The results revealed the presence of a tick bite granuloma. This case highlights the importance of removing all mouthparts left in the skin after tick removal, as this can avoid the need for further surgical intervention.

## 1. Introduction

The common occurrence of ticks (Acari: Ixodidae) in urban and suburban recreational areas has considerably increased the risk of their attacks on humans.

Pharmacologically active substances (cytolytic agents, vasodilators, platelet aggregation inhibitors, anticoagulants, anti-inflammatory proteins, and inhibitors of complement activation) secreted by the salivary glands of ticks during different phases of feeding facilitate their persistent attachment and feeding on the host and suppress the host’s innate and adaptive immune responses [1,2]. Ixodid ticks can parasitize the host for a few to over ten days, depending on the species, developmental form, and environmental conditions [3].

During feeding, ticks can transmit various tick-borne pathogens (TBPs) with their saliva to human or animal hosts. These infectious agents pose a considerable threat to public health, e.g., Lyme disease, Spotted Fever Group rickettsioses, and tick-borne encephalitis. Tick bites can also cause non-infectious complications, such as local skin lesions or systemic reactions, e.g., tick paralysis and allergy to non-primate mammalian meat, known as alpha-gal syndrome (AGS) [4].

A pivotal role in limiting the impact of ticks as vectors is attributed to the removal of intact parasites from the skin immediately after the infestation. The risk of TBP infection increases with the length of tick feeding. Upon attachment to the host’s skin, Powassan virus can be transmitted as early as after 15–30 min, *Rickettsia rickettsii* can be introduced after 2 h, and other pathogens, such as *Borrelia burgdorferi* spirochetes, can be transmitted to the host after 24–48 h [5].

The aim of this study was to describe the dermatological symptoms that develop after the incomplete removal of an *Ixodes ricinus* female tick from human skin. *Ixodes ricinus* is medically the most relevant tick species in Europe [6].

## 2. Case Presentation

**Figure 1:**
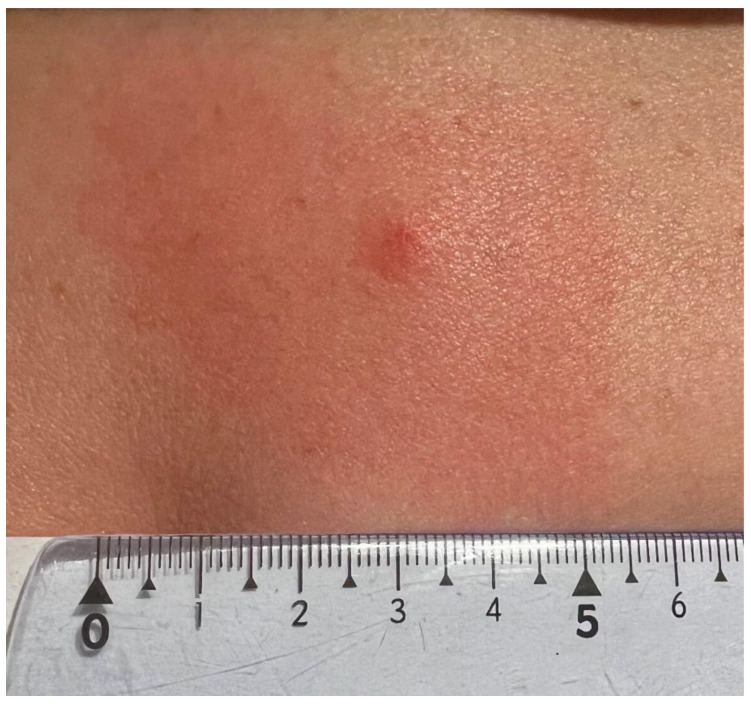
Small red solid nodule 24 h after *Ixodes ricinus* female tick removal with erythema due to skin inflammation around the tick bite area (photograph by Katarzyna Bartosik).

**Figure 2:**
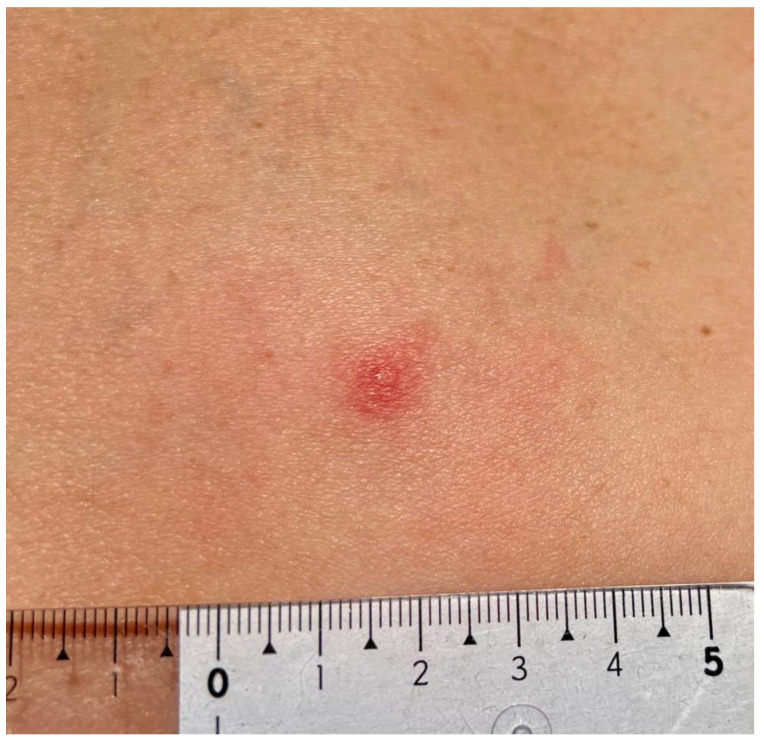
Small red solid nodule 48 h after *Ixodes ricinus* tick removal with centrally forming erosion. Mild erythema due to skin inflammation around the tick bite area (photograph by Katarzyna Bartosik).

A 47-year-old female patient, a member of the research team, reported to a dermatology clinic with a skin lesion at the tick bite site in the lumbar region 9 weeks after tick removal. An erythematous-infiltrative lesion, with a diameter of approx. 4.5–5 cm without clear demarcation from the surrounding area, developed 24 h after the removal (Figure 1). The tick feeding site was visible as an erythematous papule in the central part. The diameter of infiltration and erythema gradually decreased (Figure 2) until complete reduction after 72 h. The tick attachment site exhibited skin erosion, which did not heal for 9 weeks.

**Figure 3:**
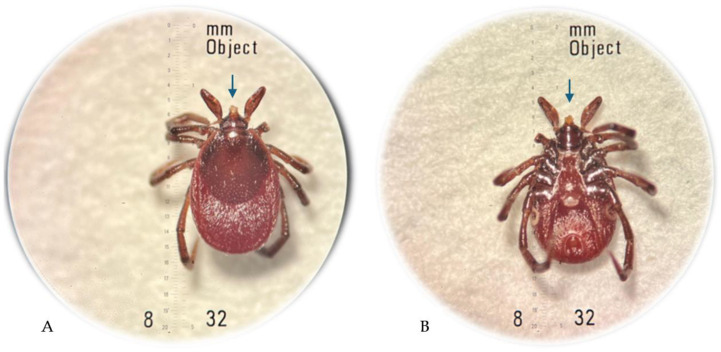
*Ixodes ricinus* female tick incompletely removed from the patient’s skin: dorsal view (**A**); ventral view (**B**), where the damaged mouthparts are indicated by an arrow; original magnification 32× (photographs by Katarzyna Bartosik).

The arthropod removed from the skin was transferred to the laboratory and preserved in 40% ethanol. The specimen was examined using a Stemi DV4/DR stereoscopic microscope (Carl Zeiss, Oberkochen, Germany) and identified as an *I. ricinus* female tick with the use of the standard taxonomic key [7]. The hypostome/mouthparts of the tick were damaged by the improper removal by hand (Figure 3). The morphological examination of the tick based on the morphometric criteria defined for *I. ricinus* females in different feeding stages [8] and the patient interview indicated no longer than 12-hour feeding of the tick before the removal.

**Figure 4:**
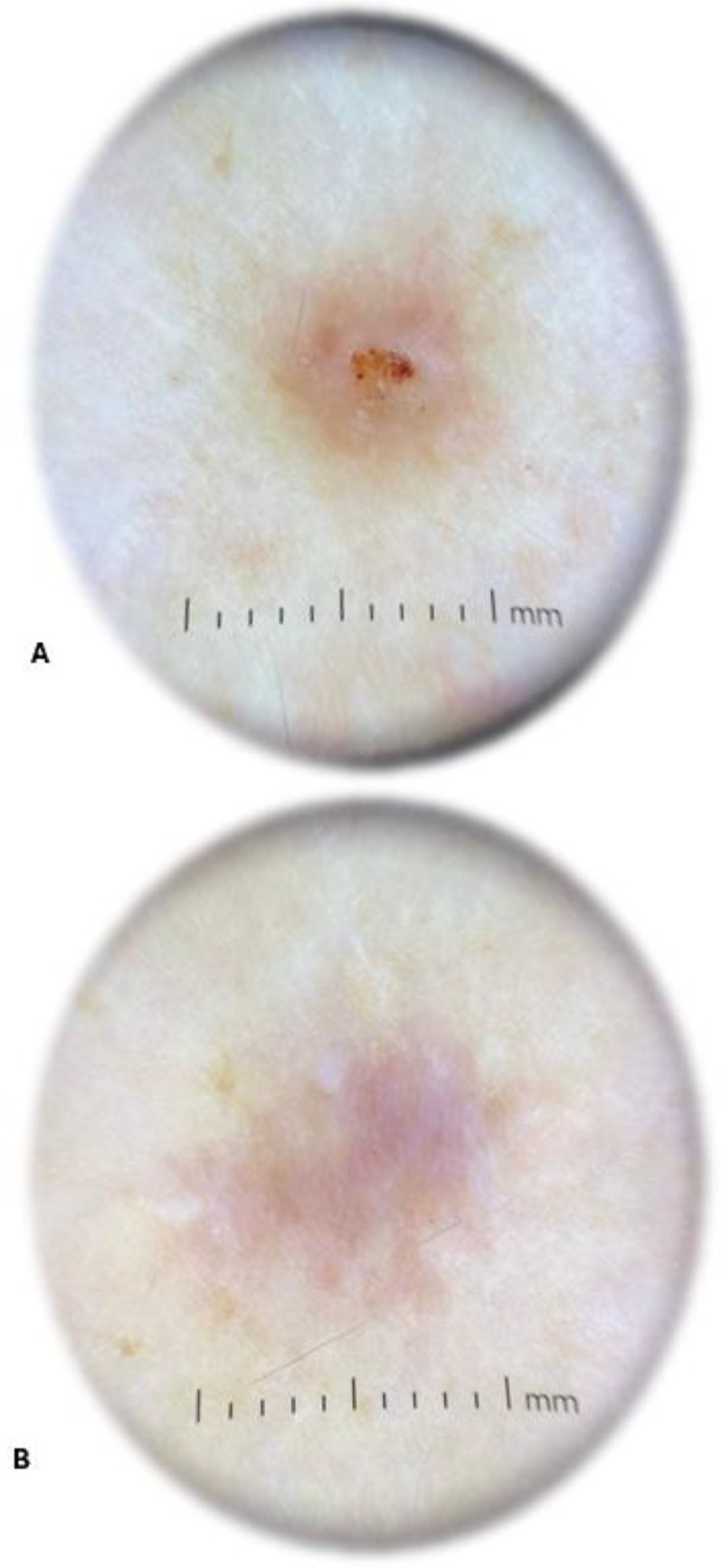
Dermatoscopic image of the lesion 9 weeks after *Ixodes ricinus* female tick removal revealing centrally located erosion covered with crust and peripheral hyperpigmentation with white linear streaks (**A**) and a scar after surgical removal of the tick bite granuloma (**B**) (photographs by Agata Szczecina).

The physical examination performed by a dermatologist showed an elevated solid purple nodular lesion with a size of 6 × 4 mm. In the dermatoscopic examination, the lesion was identified as centrally located erosion covered with crust. It exhibited peripheral hyperpigmentation and white linear streaks (Figure 4). The skin lesion caused subjective symptoms, such as persistent itching and burning, lasting from 3 days to 9 weeks after tick removal. The changed skin fragment with subcutaneous tissue (0.8 × 0.6 × 0.9 cm) with a brown lesion measuring 0.4 cm in diameter was excised surgically under local anesthesia for routine histological sectioning. The skin sample was placed in 10% buffered formalin for 6 h and processed into paraffin sections using conventional techniques, i.e., it was dehydrated through graded alcohols, embedded in paraffin blocks, cut into 4 μm thick sections, and transferred to glass slides. The sections for the histopathological examination were stained with hematoxylin and eosin (H+E).

**Figure 5:**
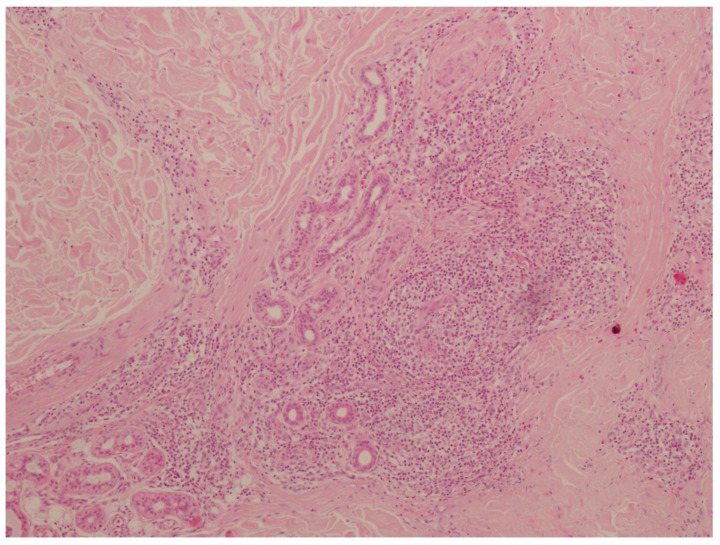
Microscopic slide in histopathological examination: a tick bite granuloma composed of lymphocytes, plasma cells, eosinophils, multinucleated macrophages, and irregular fibrosis (hematoxylin and eosin staining) (photograph by Agata Szczecina).

The histopathological examination revealed abundant inflammatory infiltrates composed of lymphocytes, plasma cells, and eosinophils located in the skin stroma, especially in the area of skin adnexa and vessels. These infiltrates comprised multinucleated macrophages. Additionally, the lesions were accompanied by irregular fibrosis (Figure 5). No elements of the tick gnathostome were found in the skin sections. They had probably been removed mechanically by the patient while scratching the tick feeding site. Slight wound exudation and desquamation were observed. The histopathological image indicated morphological reactive lesions induced by the tick bite (tick bite granuloma). After the inflammatory lesion was removed, the skin healed without complications and the symptoms of inflammation, i.e., itching and burning, resolved.

## 3. Discussion

All experimental trial-based studies highlight the importance of removing ticks intact, as this is a key factor in minimizing potential medical complications after removal. Akin Belli et al. [9] evaluated the effectiveness of various tick removal tools in their study involving 160 feeding *I. ricinus* ticks. They examined the following methods: tweezers, the lassoing technique, card detachment, and freezing. The removal efficacy rates for each method were as follows: 82.5% (33 out of 40) for tweezers, 47.5% (19 out of 40) for the lassoing technique, 7.5% (3 out of 40) for card detachment, and 0% (0 out of 40) for freezing. The differences in the efficacy among these methods were statistically significant (*p* < 0.001). It is also important to note that, while some tools are specifically designed for tick removal, they may not be effective for juvenile ticks or for adult ticks that are tightly attached. Our observations indicate that the improper removal of a tick from human skin may result in the development of persistent skin lesions causing long-lasting discomfort to the patient. To the best of our knowledge, this is the first description of such a case associated with infestation by a female of *I. ricinus* tick.

It is important to highlight the clinical long-term dermatological consequences of the incomplete removal of *I. ricinus* ticks, as females and nymphs of this species attack humans most frequently in a large part of Europe [6,10,11].

Local skin lesions in humans induced by infestations by various species of ticks have been reported repeatedly [12,13,14,15,16,17,18,19]. These reports show that the lesions may have various macroscopic pictures. Most frequently, inflammatory lesions of varying intensity and size can be observed at the tick feeding site, ranging from a punctate wound to redness and swelling with accompanying pain, inflammation of the lymphatic vessels, and enlargement of the surrounding lymph nodes. Histopathological examinations of skin sectioned from the tick attachment site confirm the inflammatory nature of skin lesions exhibiting inflammatory infiltrates with various cellular compositions characteristic of acute, subacute, or chronic inflammatory reactions to tick saliva components, papules, and nodules. A persistent reaction to tick saliva components may lead to the development of granulomatous reactions [13,20,21,22,23]. Granuloma formation is also triggered by foreign bodies, e.g., a tick hypostome fragment left in the skin [24].

The present observations indicate that reactive skin lesions can appear in humans even after a short period of tick feeding. The tick bite granuloma described in this case report may have developed as a result of a reaction to the components of saliva of the *I. ricinus* female tick removed from the skin already in the first feeding phase. It may also have been a reaction to the tick’s mouthparts that had not been fully removed from the skin.

In addition to the stimulation of inflammatory reactions, the tick’s mouthparts left in the skin together with parts of the cement cone may enhance the risk of human infection with some tick-borne pathogens, such as tick-borne encephalitis viruses, contained in the cement substance [25]. The cement cone is secreted around the hypostome inserted in the host’s skin by most ixodid ticks, including *I. ricinus* [23].

The knowledge of the immunomodulation induced by tick saliva during feeding is still incomplete. In their studies, Glatz et al. [26] have shown that the local immune response to *I. ricinus* tick bites varies, depending on the length of tick attachment to human skin. Early tick bite lesions in humans are dominated by macrophages and dendritic cells with elevated mRNA for macrophage and neutrophil chemoattractants, as well as pro-inflammatory cytokine IL-1β and anti-inflammatory cytokine IL-5. In turn, lesions induced by longer tick parasitism periods exhibit an increased number of lymphocytes and a reduced number of macrophages and neutrophils. The weaker response triggered by 24-hour tick feeding is probably associated with the immunomodulatory effect of tick saliva.

The intensity of histological and cytological lesions in the host’s skin may be influenced by the morphological and physiological features of the tick (hypostome length and skin penetration depth and composition of saliva changing in different feeding phases), the duration of tick attachment to the host’s skin, and the host’s immunity [4,23,26].

After proper removal of an *I. ricinus* tick, skin lesions may not appear at all or may persist for up to 48 h, fading gradually within a few days [12].

## 4. Conclusions

The intense and persistent skin reaction that develops after the improper removal of the tick attached to the skin for a short time suggests the need to inform patients about the recommended tick removal methods. In the event of tick damage during removal from the skin, the tick’s mouthparts should be removed from the skin immediately after the bite. The removal of ticks intact can minimize the effects of their feeding, e.g., allergy, anaphylaxis, or TBP transmission, and help to avoid the need for surgical intervention.

## Data Availability

The original contributions presented in this study are included in the article, and further inquiries can be directed to the corresponding author.
